# Modified 3D Graphene for Sensing and Electrochemical Capacitor Applications

**DOI:** 10.3390/nano14010108

**Published:** 2024-01-02

**Authors:** Kavitha Mulackampilly Joseph, Gabrielle R. Dangel, Vesselin Shanov

**Affiliations:** 1 Department of Mechanical and Materials Engineering, University of Cincinnati, Cincinnati, OH 45221, USA; josephka@mail.uc.edu; 2 Department of Chemistry, University of Cincinnati, Cincinnati, OH 45221, USA; dangelgr@mail.uc.edu; 3 Department of Chemical and Environmental Engineering, University of Cincinnati, Cincinnati, OH 45221, USA

**Keywords:** 3D graphene, nitrogen-doping, defective, square wave anodic stripping voltammetry (SWASV), Electric Double Layer Capacitor (EDLC)

## Abstract

Less defective, nitrogen-doped 3-dimensional graphene (N3DG) and defect-rich, nitrogen-doped 3-dimensional graphene (N3DG-D) were made by the thermal CVD (Chemical Vapor Deposition) process via varying the carbon precursors and synthesis temperature. These modified 3D graphene materials were compared with pristine 3-dimensional graphene (P3DG), which has fewer defects and no nitrogen in its structure. The different types of graphene obtained were characterized for morphological, structural, and compositional assessment through Scanning Electron Microscopy (SEM), Raman Spectroscopy, and X-ray Photoelectron Spectroscopy (XPS) techniques. Electrodes were fabricated, and electrochemical characterizations were conducted to evaluate the suitability of the three types of graphene for heavy metal sensing (lead) and Electric Double-Layer Capacitor (EDLC) applications. Initially, the various electrodes were treated with a mixture of 2.5 mM Ruhex (Ru (NH_3_)_6_Cl_3_ and 25 mM KCl to confirm that all the electrodes underwent a reversible and diffusion-controlled electrochemical process. Defect-rich graphene (N3DG-D) revealed the highest current density, followed by pristine (P3DG) and less-defect graphene (N3DG). Further, the three types of graphene were subjected to a sensing test by square wave anodic stripping voltammetry (SWASV) for lead detection. The obtained preliminary results showed that the N3DG material provided a great lead-sensing capability, detecting as little as 1 µM of lead in a water solution. The suitability of the electrodes to be employed in an Electric Double-Layer Capacitor (EDLC) was also comparatively assessed. Electrochemical characterization using 1 M sodium sulfate electrolyte was conducted through cyclic voltammetry and galvanostatic charge-discharge studies. The voltammogram and the galvanostatic charge-discharge (GCD) curves of the three types of graphene confirmed their suitability to be used as EDLC. The N3DG electrode proved superior with a gravimetric capacitance of 6.1 mF/g, followed by P3DG and N3DG, exhibiting 1.74 mF/g and 0.32 mF/g, respectively, at a current density of 2 A/g.

## 1. Introduction

Three-dimensional graphene (3DG) has been attracting increasing attention from academia and research due to its unique properties, such as high electrical conductivity, large specific surface area, excellent porous network structure, high mechanical strength, and flexibility. This material can overcome the aggregation or stacking of graphene flakes, in addition to inheriting the properties of 2D graphene. 3D graphene synthesized by Chemical Vapor Deposition (CVD) has a monolithic structure with low defects and high purity [1,2] and can be tailored to alter its physical and electrochemical properties based on the type of bonding, heteroatom doping, surface functional group, and defect engineering. Due to the above reasons, graphene is considered a potential candidate in cutting-edge applications like energy conversion/storage and electrochemical and biosensing [3,4,5,6,7,8].

One characteristic that makes graphene ideal for sensing applications is the homogenous distribution of electrochemically active sites on a nanometer scale and the large potential window. These advantages result in superior electrochemical activity, quicker electron and ion transport, and reduced charge-transfer resistance. Moreover, the unique properties of graphene, such as its huge surface area and high electrical conductivity, help it to efficiently attach to the analyte molecule, resulting in higher sensitivity and a better signal-to-noise ratio in graphene-based sensors [9]. Hence, such devices can sensitively detect heavy metal ions like lead in drinking water. 

Lead is toxic and a serious threat to the environment, aquatic ecosystems, and human beings. Therefore, the detection of lead is important [2]. A powerful and popular electrochemical technique called anodic stripping voltammetry (ASV) is used for lead detection in aqueous solutions. This technique involves an initial preconcentration step wherein the lead is allowed to accumulate on the electrode surface by reduction from its ionic state into its atomic state, followed by a stripping step wherein the metal is re-oxidized to its ionic form. ASV is a quick, simple, portable, inexpensive, and highly sensitive technique with low detection limits. Efficient electrodes are paramount for ASV. Mercury was widely used due to its reproducibility and sensitivity, but its toxicity and necessity for adequate disposal limited its usage. Bismuth was used as a replacement electrode for mercury, but its narrow voltage window limits it from detecting Hg and Cd. Hence, research on advanced electrodes like nanocarbons, especially graphene, is in progress in the quest for a stable electrode with a wide voltage window [10,11,12].

One other cutting-edge application in which graphene is an ideal candidate due to the aforementioned unique properties is its employment as an Electric Double-Layer Capacitor (EDLC) electrode. The charge storage mechanism in EDLC is the formation of a double layer of electrolyte ions on the conductive electrode surface, a physical process that does not involve any chemical reactions. Hence, EDLCs are not restricted by the redox reaction kinetics as in batteries and can be charged and discharged quickly a million times. However, they have the drawbacks of low capacitance and low energy density [13]. The tunability of graphene by doping/surface functionalization, etc., is one of the strategies adopted to overcome this disadvantage. 

Heteroatom doping induces structural modification, which can control the electrochemical properties of graphene. Several studies have proven that nitrogen, oxygen, and boron doping can change the sp^2^/sp^3^ ratio of carbon-based electrodes [6]. The capability of nitrogen to alter the charge carrier density of graphene results in a modification of its electronic structure. This can enhance the capacitance and energy density of graphene electrodes in both aqueous and organic electrolytes, which has been investigated and confirmed [13].

In this work, three types of CVD-synthesized 3-dimensional graphene, differing in their sp^2^/sp^3^ ratio, nitrogen doping content, and oxygen presence, are compared for their electrochemical response in lead sensing and EDLC applications. The three types of graphene are (i) pristine or P3DG, which is undoped, less defective (high sp^2^), and with low oxygen content (1.5%); (ii) N3DG or nitrogen-doped, less defective (high sp^2^), and with medium oxygen content (8–9%); and (iii) N3DG—D or nitrogen-doped with high defect (low sp^2^) and high oxygen (20%) content. By comparing the three types of graphene, this work aims to confirm the positive effects of (a) high sp^2^ and less defective structure, (b) nitrogen doping, and (c) reveal the type of oxygen-containing groups that can help with lead sensing and EDLC applications.

## 2. Materials and Methods

### 2.1. Materials 

Three-dimensional Graphene (3DG) is synthesized using nickel catalyst slurry. To make this slurry for graphene synthesis, the following chemicals were employed: nickel powder with 3–7 μm particle size and 1.8–2.7 g/cm^3^ apparent density (Alfa Aesar, Tewksbury, MA, USA), toluene (Sigma Aldrich, Milwaukee, WI, USA), polystyrene (21,000 Mw), plasticizer di-(ethylene glycol) dibenzoate (DEGD), (TCI SG 1.18), acetonitrile (99.0%) and ionic liquid EMIMBF4 (99.0%), both from Sigma-Aldrich Co Ltd. The gases in the CVD process were ultra-high purity grades (UHP), like CH_4_, H_2_, and Liquified Argon, which were procured from the Wright Brothers, Cincinnati, OH, USA. All the chemicals were used as received. 

For lead sensing, the following chemicals were employed: potassium chloride (KCl, 99%), nitric acid (70%, trace metal basis), sodium acetate trihydrate (BioXtra, ≥99.0%), and Pb (NO_3_)_2_ standard solution (99.95–100.5%, Trace CERT ICP standard grade), all purchased from Sigma-Aldrich (St. Louis, MO, USA). Hexamine ruthenium (III) chloride (Ru (NH_3_)_6_Cl_3_ or Ruhex, 98%), used as a reversible redox molecule, was purchased from Acros Organics (Waltham, MA, USA). Glacial acetic acid was purchased from Pharmco (Shelbyville, KY, USA). All aqueous solutions employed Milli-Q ultra-pure water (18.2 MΩ cm). Acetate buffer (0.1 M, pH 4.5) was prepared from sodium acetate trihydrate, glacial acetic acid, and Milli-Q ultra-pure water. The lead stock solution was made in a 2% nitric acid solution.

### 2.2. Experimental Methods

#### 2.2.1. Synthesis of 3-Dimensional Graphene (Pristine-P3DG), Nitrogen-Doped Graphene (N3DG), and Nitrogen-Doped-Defective Graphene (N3DG-D)

**Figure 1:**
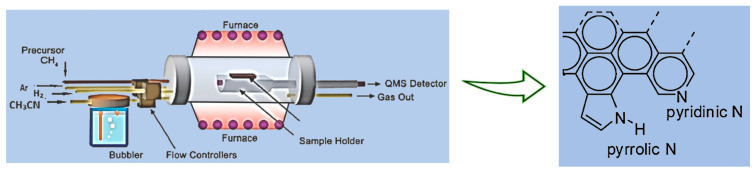
Schematic of CVD synthesis of nitrogen-doped graphene. The CVD process (**left**) and structure of N-doped graphene (**right**).

The synthesis of pristine three-dimensional graphene (P3DG) via CVD is detailed in our publication elsewhere [14]. The same method was followed for synthesizing nitrogen-doped less defective graphene (N3DG), except for a slight modification of using acetonitrile as the nitrogen dopant [15]. The detailed process is as follows: as nickel was the catalyst for graphene synthesis, 40 g of Ni powder and 20 mL of toluene were mixed with a pre-prepared polystyrene (5.5 g) solution in 20 mL toluene. The above mix was knife-casted and air-dried for 24 h, then cut into specified dimensions. The cut Ni-polymer catalyst substrate was introduced into the CVD reactor (ET 1000, First Nano Make), which had the preprogrammed recipe for graphene synthesis. The furnace temperature was ramped up from room temperature to 1000 °C at a rate of 25 °C/min in 40 min, simultaneously passing H_2_ (325 sccm) and Ar (1000 sccm) into the reactor. The temperature was held at 1000 °C for five more minutes to ensure the sintering of the Ni particles and complete removal of the polymer. This was followed by the synthesis step, where the carbon precursor gas methane (25 sccm) and acetonitrile (20 sccm) as a nitrogen dopant were introduced for 5 min, followed by a rapid cooling step at a rate of 100 °C/min at the presence of H_2_ (325 sccm) and Argon (1000 sccm). Figure 1 illustrates a schematic of the CVD process and the obtained nitrogen-doped graphene structure.

The cooled samples were retrieved from the CVD reactor, and the synthesized N3DG graphene with Ni particles was immersed in an acid bath containing 3 M HCl for 10 h at 80 °C to remove the metal catalyst. The Ni-free graphene was rinsed in D.I. water for 3–4 h, followed by ethanol for 30 min, and air dried at room temperature for 24 h to obtain high-quality N3DG.

High-defect, nitrogen-doped graphene (N3DG-D) was synthesized similarly except for using acetonitrile as both precursor and dopant, without methane, and employing a lower reaction temperature, as shown in Table 1.

#### 2.2.2. More Details on Graphene Synthesis from Methane and Acetonitrile

Chemical vapor deposition practiced in this work for the synthesis of graphene included two steps. The first one enables the thermal decomposition of the carbon precursor methane in the presence of hydrogen and argon, followed by the dissolution of the carbon atoms into the nickel catalyst. The second step is simply quenching the Ni catalyst with the dissolved carbon atoms in it, which causes their precipitation, thus forming graphene on the catalyst surface [16]. Further details can be found in our publication [14].

While gaseous precursors are common for CVD synthesis of graphene, liquid precursors like hexane, benzene, and alcohols such as propanol, ethanol, and methanol are also used as alternatives to gaseous precursors. Acetonitrile is frequently employed in graphene synthesis as it offers a simple and effective way of nitrogen doping. Another advantage of this easy-to-handle liquid precursor is that it enables a low synthesis temperature [17]. Acetonitrile facilitates the growth of multilayer graphene on the nickel catalyst surface. It decomposes into CH_3_ and CN-containing functional groups, which can increase the rate of graphene growth and simultaneously realize nitrogen doping within its crystal lattice [18]. The N-doped graphene formed from acetonitrile reveals a reasonably higher quality, confirmed by Raman spectroscopy and XPS analysis detailed in the following sections. The formation of graphene from acetonitrile is proven to occur by its direct decomposition, as described in [19].

#### 2.2.3. Characterization and Measurements 

The mass of the graphene electrodes was precisely measured with a Model ME5 Sartorius micro-analytical balance having microgram resolution. X-ray photoelectron Spectroscopy (XPS) was conducted using a V.G Thermo Scientific Multi Lab 3000 ultra-high vacuum surface analysis system, with a base pressure of 10^−9^ Torr and an Al Kα as electron source excitation energy. Surface imaging of graphene was obtained through FEI SCIOS dual beam 5 kV Scanning Electron Microscopy. A Renishaw, in Via instrument, collected the Raman spectra of graphene using a 514 nm Ar-ion laser spot size = 1 μm^2^.

#### 2.2.4. Electrochemical Characterization for Lead Detection 

Cyclic voltammetry (CV) and square wave anodic stripping voltammetry (SWASV) were performed on a CH Instruments electrochemical workstation (CHI760E, Austin, TX, USA). To determine the charging current of the electrodes, CVs were run in 0.1 M acetate buffer (pH 4.5) with a scan rate of 0.1 V/s from −0.1 V to +0.1 V. 

All electrodes were tested in 50 mM KCl from +0.4 V to +0.1 V with a scan rate of 0.05 V/s and then in a solution containing 2.5 mM Ruhex, a reversible redox molecule, and 25 mM KCl. A scan rate study was performed on N3DG-D, N3DG, and P3DG in a solution of 2.5 mM Ruhex and 25 mM KCl, where the scan rates used were 0.001, 0.005, 0.007, 0.01, 0.03, 0.05, 0.07, 0.1, 0.125, 0.15, and 0.2 V/s. This was conducted to determine whether the electron transfer was a diffusion or absorption-controlled process. 

Finally, lead detection was performed using SWASV. Initially, blanks were collected in 0.1 M acetate buffer (pH 4.5). Then, 1.5 μM Pb^2+^ standard was spiked into the acetate buffer to be detected. A preconditioning or cleaning step was applied first, where the potential was held at 0.1 V for 60 s. Then, a deposition potential (E_dep_) of −1.2 V was applied for 180 s, allowing Pb^2+^ to reduce onto the electrode as Pb^0^. The potential was then swept from −1.2 V to +0.3 V with an amplitude of 0.025 V, a frequency of 5 Hz, and a potential step (Estep) of 0.005 V to oxidize Pb^0^ back to Pb^2+^. 

#### 2.2.5. Electrochemical Characterization for EDLC Performance 

Electrochemical measurements for EDLC characterizations such as cyclic voltammetry (CV), galvanostatic charge–discharge (GCD), and electrochemical impedance spectroscopy (EIS) were conducted with a workstation Gamry, Interface 1000. 1 M sodium sulfate as an electrolyte was used on the three-electrode setup with Ag/AgCl as the reference electrode and Pt wire as the counter electrode. EIS measurements were recorded from 10 kHz to 10^−1^ Hz, applying a sinusoidal voltage amplitude of 10 mV at the open circuit potential. The electrode design and the test setup are given in our previous publication [20]. Electrochemical calculations for EDLC assessment are as follows.

The capacitance *C* of the electrode was calculated from the GCD curves using the equation [21,22]:

(1) $$\mathrm{Gravimetric}\;\mathrm{capacitance}\;Cm=\frac{I}{m}*I\Delta t/∆V$$

where *I* is the discharge current, *t* is the discharge time, ∆V is the working voltage window, and *m* is the device’s mass. 

For non-linear GCDs,

(2) $$\mathrm{Gravimetric}\;\mathrm{capacitance}\;Cm=\frac{I}{m}\int 1/V(t)\cdot dt.$$

where *I* is the applied constant current density, *t* is the discharge time, and *V*(*t*) is the potential as a function of *t*.

## 3. Results and Discussions 

### 3.1. Surface Characterizations 

#### 3.1.1. Morphology 

**Figure 2:**
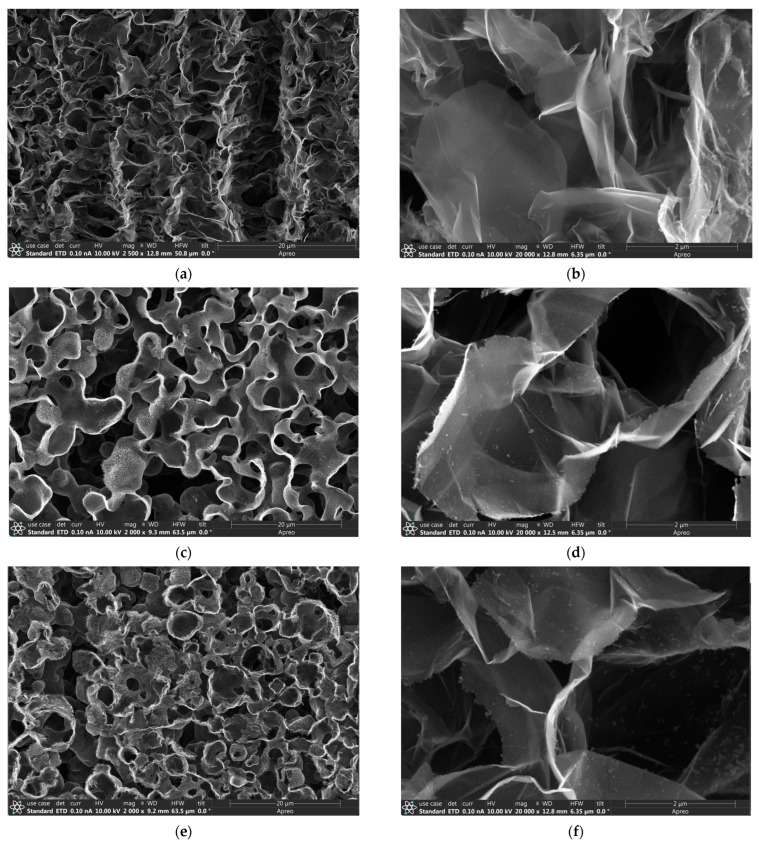
Morphology of three types of graphene. SEM images of pristine (**a**,**b**), nitrogen-doped (**c**,**d**), and defective nitrogen-doped graphene (**e**,**f**). Images (**a**,**c**,**e**) are taken at low magnification, and (**b**,**d**,**f**) reveal high-magnification images.

Figure 2 displays the morphology of the three types of graphene studied by SEM. The SEM images of the P3DG, N3DG, and N3DG-D are given in Figure 2a–f. Figure 2a,c,e represent low magnification images, while those in Figure 2b,d,f are taken at high magnification. All the SEM images exhibit hierarchically interconnected porous network morphology, which enhances the electrochemical process by facilitating electrolyte infiltration into the graphene sheets while minimizing their restacking. The porous structure also imparts high flexibility along with mechanical integrity. One of the inherent properties of CVD-grown graphene, interconnected by 3D scaffold structure, is the presence of wrinkles/ripples at the grain boundaries of graphene flakes [2]. The reason for the wrinkles is the difference in the thermal expansion coefficient between nickel and graphene. 

Both the low defect (Figure 2c,d) and high defect (Figure 2e,f) nitrogen-doped graphene exhibit more open and well-defined pores with curved and closed edges compared to pristine graphene (Figure 2a,b). Nitrogen doping creates more active sites in the graphene lattice, shifting the Fermi level above the Dirac point [23], and ultimately resulting in enabling charge transfer and storage. Also, the variance in surface morphology can result in Fermi energy differences between the three types of graphene, which can cause modifications in surface reactions [24].

#### 3.1.2. Raman Spectroscopy 

**Figure 3:**
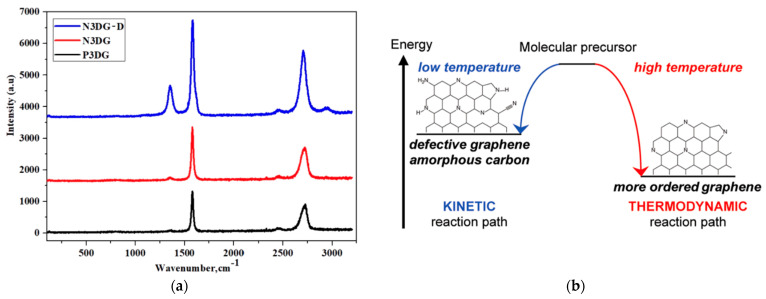
(**a**) Raman Spectroscopy of three types of 3D graphene; (**b**) schematic of kinetically and thermodynamically controlled reaction pathways for the formation of N-doped graphene. Adapted with permission from [19].

Figure 3 presents the Raman spectra of 3 types of 3D graphene along with the schematic of kinetically and thermodynamically controlled reaction pathways formation of N-doped graphene. All three types of graphene displayed in Figure 3a exhibit their characteristic G band and 2D band. The G peak at 1560 cm^−1^ indicates the graphitic band in symmetry. It appears due to the vibration of the sp^2^ hybridized ‘C’ atom on the same plane. 

A double resonant Raman scattering of second order is the reason for the 2D peak at 2690 cm^−1^. The ‘D’ peak indicating defects or disorder of the graphene is prominent only in N3DG-D. The influence of CVD synthesis temperature on the graphene structure and its impact on the Raman spectra can be interpreted via the schematic of Figure 3b as follows. The synthesis at low temperatures, such as 600 °C, gives rise to amorphous carbon. In contrast, at 800 °C, more defect-rich (N-doped) graphene is formed. Higher synthesis, such as 1000 °C, results in less-defective graphene. The D band in defect-rich N3DG-D can also be due to abundant oxygen functional groups that can disrupt the planar sp^2^ structure. For example, the atomic composition of N3DG-D is 20.2% O and 79.1% carbon, confirmed by XPS, as described in Section 3.1.3.

The required different synthesis temperatures are due to the difference In the activation energies needed for the precursor decomposition, resulting in carbon formation and deposition. The carbon deposits can undergo reconstruction at higher temperatures into a more ordered and stable structure due to a thermodynamically controlled process. In contrast, the kinetic reaction path is favored by low-temperature synthesis [19]. In P3DG, the D band at 1360 cm^−1^ is hardly seen, whereas in N3DG, there is a slight bump at the D band position due to limited nitrogen doping and a highly ordered, less defective graphene structure [25,26,27].

#### 3.1.3. X-ray Photoelectron Spectroscopy 

Elemental compositional analysis of the three types of graphene was assessed by X-ray Photoelectron Spectroscopy. As given in Table 2, N3DG-D has the least carbon content (79.1%), followed by N3DG (89.31%) and P3DG (98.5%). The XPS results of P3DG and N3DG are borrowed from our previous publication [20]. 

A high oxygen % is also revealed in both nitrogen-doped graphene types, although this is unintentional. Oxygen presence can be due to two reasons: (a) the oxidation of graphene sheets in the air [28] and (b) the introduction of gas impurities or system leaks during the CVD process, which happens under ambient/reduced pressure conditions [19]. Contrary to expectations, the N% is low in the case of N3DG-D (0.7%). This could be due to the gas-phase bond dissociation of C–N into carbon and nitrogen radical species. Due to their low bond dissociation energy, the latter is common in aliphatic nitrogen-containing precursors like acetonitrile. Another reason for lower nitrogen concentration could be the additional hydrogen (325 sccm) present during the synthesis, which acts as an etchant for both carbon and nitrogen [19].

**Figure 4:**
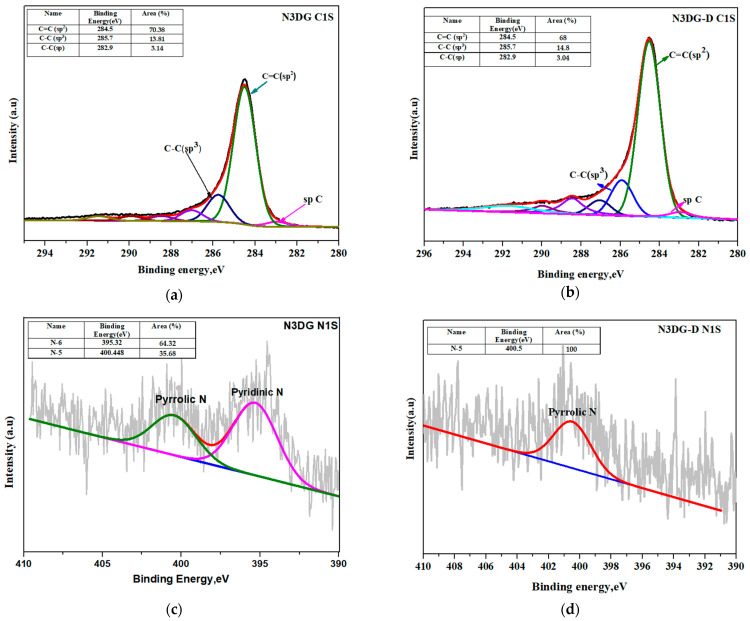

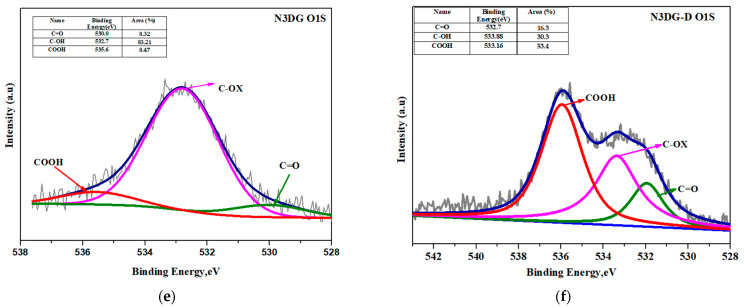
XPS high-resolution C1S of N3DG and N3DG-D (**a**,**b**), high-resolution N1S of N3DG and N3DG-D (**c**,**d**), high-resolution O1S of N3DG and N3DG-D (**e**,**f**). Figures (**a**,**c**,**e**) are adapted with permission from our previous publication [20].

Shirley-type baseline was used for performing XPS peak deconvolution along with Lorentzian (20%)/Gaussian functions. Figure 4a,b give the carbon C1s deconvolution spectra of N3DG and N3DG-D. N3DG exhibits higher sp^2^ (C–C) linkage and low sp^3^ (C–C) linkage at binding energies of 284.5 eV and 285.7 eV compared to N3DG-D [29].

The deconvoluting N1s spectra in Figure 4c,d are related to less defective N3DG and defect-rich N3DG-D. These figures revealed the presence of both pyridinic and pyrrolic linkages for N3DG at a binding energy of 395.3 eV and 400.4 eV, respectively. At the same time, high defect N3DG-D showed only pyrrolic nitrogen. The nitrogen functional group enhances the electrical conductivity by transferring electrons to the delocalized pi-conjugated system. Pyridinic nitrogen is a six-membered ring and is sp^2^ hybridized into two carbon atoms. It donates one pi electron to the aromatic system, whereas pyrrolic nitrogen is a five-membered ring donating two pi electrons and is bonded to the carbon atom of the phenolic/carbonyl group [13].

Figure 4e,f presents the high-resolution spectra of the O1s. The deconvolution of N3DG-D O1s indicated the presence of the predominant oxygen group as the carboxyl group at the binding energy of 536 eV, followed by the hydroxyl group at 533.8 eV and the carbonyl group at 532.7 eV. In contrast, the less defective N3DG showed hydroxyl groups in a greater percentage (83.21%), followed by less than 10% of carboxyl and carbonyl groups. The presence of an abundant hydroxyl group in N3DG can enhance wettability with aqueous electrolyte solutions [30,31,32], which is favorable for the applications proposed in this study.

### 3.2. Electrochemical Characterizations for Lead Detection

#### 3.2.1. Electrochemical Characterization of Electrodes

The active area of the electrodes, which is the area of the electrode where electrochemistry can occur, was determined by capacitance measurements. The cathodic and anodic peak current separation is greater than −59 V for all three graphene electrodes, reported in Table 3, meaning they are quasi-reversible processes. Initially, the charging current (*i_c_*) was determined by running a CV experiment in 0.1 M acetate buffer (pH 4.5) at a scan rate (*ν*) of 0.1 V/s. Then, using Equation (3), the double-layer capacitance (*C_dl_*) was calculated. Equation (4) was used to determine the active area of the electrode.

(3) $$i_{c}=C_{dl}\nu$$

(4) $${\;C}_{dl}=\varepsilon_{0}\varepsilon_{r}\frac{A}{d}$$

where *ε*_0_ is the permittivity of free space (8.854 × 10^−12^ F/m), *ε_r_* is the dielectric permittivity of the solution with the value of 6.15, *A* is the active area of the electrode (m^2^), and *d* is the diffusion layer thickness (m), 9.6 × 10^−10^ m. Table 3 compares the active areas between the three electrodes, where N3DG has the highest active area, followed by P3DG and N3DG-D. 

The active area is due to the higher intrinsic conductivity contributed by both nitrogen doping and sp^2^ hybridized carbon. N3DG has a comparatively higher nitrogen content and sp^2^ hybridization. N3DG-D reveals the lowest sp^2^ hybridized carbon and a small % of nitrogen content (0.7%), which results in the lowest active area. 

**Figure 5:**
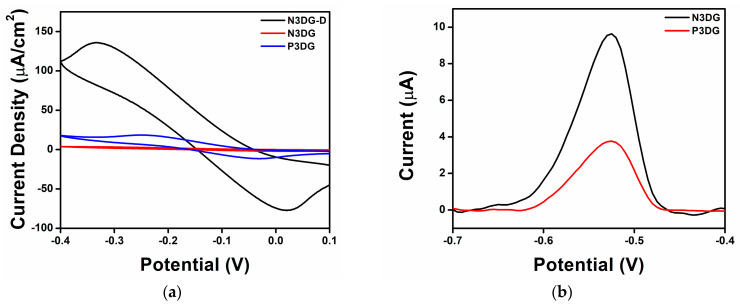
(**a**) Current Density vs. Potential for graphene electrodes in 2.5 mM Ruhex and 25 mM KCl; (**b**) Detection of 1.5 μM Pb^2+^ in 0.1 M acetate buffer.

Initially, CVs were collected in 50 mM KCl for N3DG-D, N3DG, and P3DG, shown in Figure S1, to ensure no contamination peaks were present. The electrodes had different geometrical areas; therefore, the CVs were normalized by dividing the current by the active areas to obtain the current density. Then, in a Ruhex solution, the electrodes were tested, as shown in Figure 5a. 

Comparing the CVs obtained in the KCl and Ruhex solutions, it can be found that N3DG-D showed the highest current density, followed by P3DG and N3DG, which revealed the lowest value. This indicates that N3DG has a lower charging current, which could improve the electrode’s performance. Figure S2A–C provides better visual images of the shapes of the CVs compared to their performance in 50 mM KCl. Figure S2A,C, for N3DG-D and P3DG, respectively, showed oxidation and reduction peaks, while N3DG in Figure S2B revealed a less-defined reversible redox system. The largest active area is N3DG, followed by P3DG and N3DG-D. Contrary to the result in Table 3, where the greatest active area is for N3DG, followed by P3DG and N3DG-D, in Figure 5a, the largest charging current is for N3DG-D, followed by P3DG and N3DG. This could be due to the presence of the highest percentage of oxygen functional groups in N3DG-D (20.2% as confirmed by XPS), which could contribute to pseudo-capacitance, resulting in a higher charging current. In both N3DG and P3DG, the oxygen group percentage is less than 10%.

All the tested electrodes proved that electron transfer is a diffusion-controlled process. The latter was determined through a scan rate study in the Ruhex solution. Figures S3–S5 display the graphs representing these experiments where a linear response is seen when the current versus the square root of the scan rate is plotted. The linear reactions are reported for the cathodic and anodic peak currents in Equations (S1)–(S6) for these electrodes.

#### 3.2.2. Lead Detection 

SWASV was used to detect dissolved lead. The parameters employed for the analysis were taken from our previous work [2], where graphene foam electrodes were used to detect lead in acetate buffer, and the lowest concentration tested was 1.5 μM. In the current work, lead detection was used to compare the electrodes and determine which had the most promising performance for lead detection. The electrodes were tested in 0.1 M acetate buffer only, and the obtained results are illustrated with black curves in Figure S6A–C. Then 1.5 μM Pb^2+^ was spiked into acetate buffer, which yielded the red curves in Figure S6A–C. The N3DG-D response in Figure S6A showed a prominent peak from around 0.8 V to −0.3 V in the background current, and when the lead was added, that peak continued to increase in both width and height. The stripping peak for lead was around −0.5 V. However, due to the broad peak in the N3DG-D voltammogram, it was challenging to attribute this increase to lead. 

In general, graphene-based sensors have a higher specific surface area with greater active sites that increase sensitivity. Nevertheless, they can also cause increased capacitive or background current, resulting in a poor signal-to-noise ratio and reducing the detection limit. Moreover, in the case of N3DG-D, defects and oxygen functional groups on its surface can change its electronic properties, which might also slow down the electrode activity [6]. However, in Figure S6B, for N3DG, and Figure S6C for P3DG, no peak is seen around −0.5 V when tested in only an acetate buffer. Then, when conducting the test in the 1.5 μM Pb^2+^ acetate buffer solution, a peak for lead can be seen at −0.5 V. Figure 5b shows the lead peak from N3DG (black) and P3DG (red) in the same graph. N3DG showed better sensitivity to the lead with a peak height of 9.44 μA and a peak area of 0.77 V·μA, whereas P3DG revealed a peak height of 3.83 μA and a peak area of 0.28 V·μA, as reported in Table 4. The results of this comparison demonstrated that the N3DG electrode was better for detecting lead than the P3DG one. 

To summarize the lead detection results, the three types of graphene electrodes tested for lead sensing showed significant differences in their electrochemical characterization and lead detection response when using acetate buffer. N3DG-D revealed the largest charging current and smallest active area of the three electrodes tested. Due to a large background current peak in the SWASV for acetate buffer solution, no lead peak is seen when testing in 1.5 μM Pb^2+^. The inability of the N3DG-D electrode to detect lead in this case is attributed to its large charging current and small active area. N3DG showed the smallest charging current and the largest active area. Finally, the P3DG electrode revealed the charging current and active area between N3DG-D and N3DG. It was found that N3DG and P3DG could detect 1.5 μM Pb^2+^ in acetate buffer; however, N3DG showed a higher peak current and peak area. This indicated that N3DG was more sensitive to lead detection than P3DG, making it the most promising electrode among the three tested for lead detection. 

### 3.3. Electrochemical Characterizations for EDLC Properties

**Figure 6:**
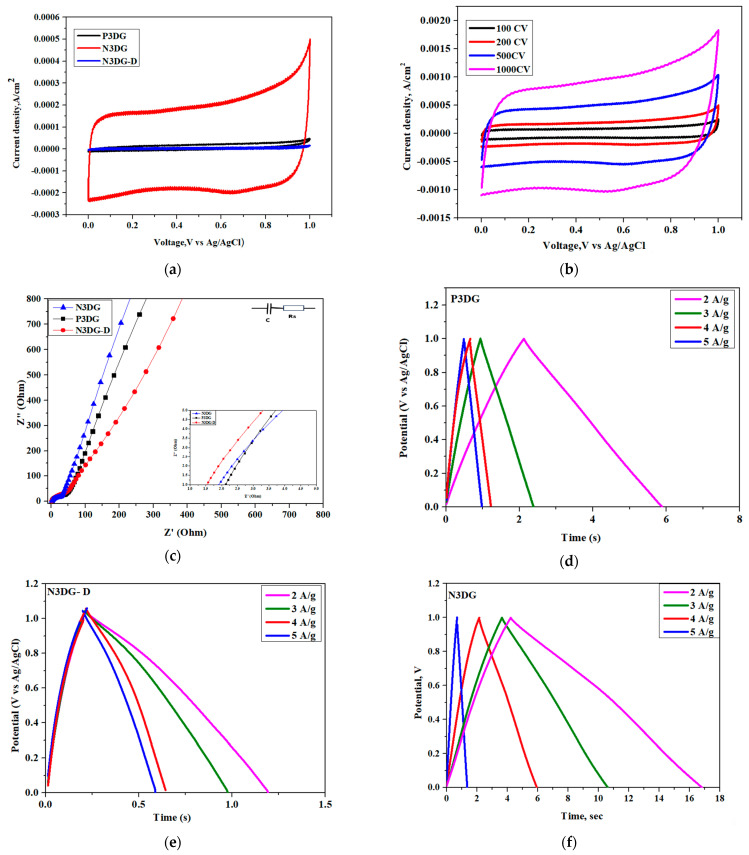

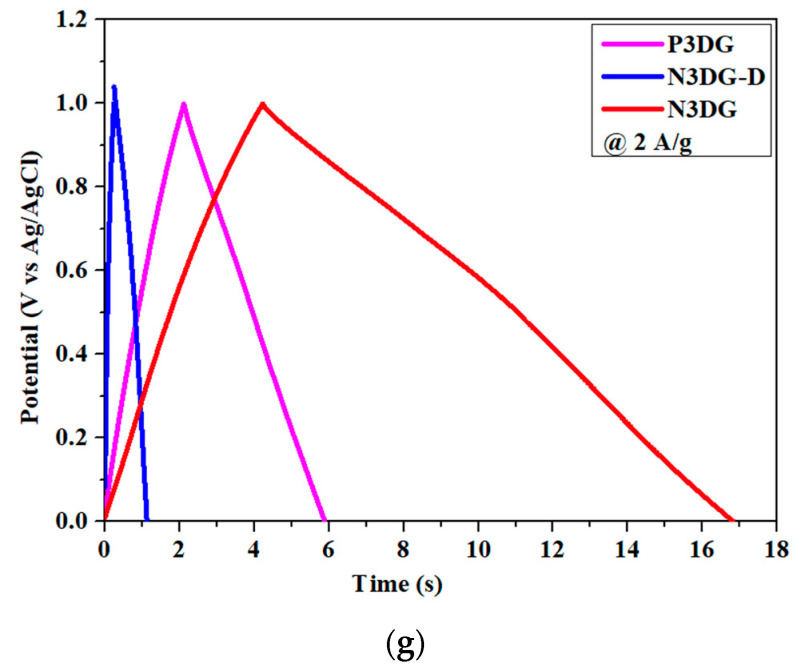
(**a**) CV curve comparison of P3DG, N3DG, and N3DG-D; (**b**) Scan rate study of N3DG; (**c**) EIS comparison of P3DG, N3DG, and N3DG-D; (**d**–**f**) GCD curves at different current densities for P3DG (**d**), N3DG-D (**e**) and N3DG (**f**); (**g**) GCD comparison of P3DG, N3DG, and N3DG-D at a current density of 2 A/g.

The three types of electrodes were also assessed for Electric Double-Layer Capacitor (EDLC) behavior using electrochemical measurements. This was conducted in a three-electrode setup, with Ag/AgCl as the reference electrode and platinum as the counter electrode. P3DG, N3DG, and N3DG-D were used as working electrodes separately. Figure 6a displays the comparative cyclic voltammograms of the three electrodes at a scan rate of 200 mV/s. 

The less defective N3DG had the largest voltammogram area compared to the other two. The CV profile is quasi-rectangular for all three graphene types without any Faradaic reaction, indicating that the charge storage mechanism is due to surface reactions. This also implies that the process is not diffusion-controlled, and the adsorption/desorption occurs at the electrode-electrolyte interface, indicating the suitability for EDLC/supercapacitor application. 

The CV plot comparative area of the three electrodes is in agreement with the active area calculation for these electrodes following the sequence N3DG > P3DG > N3DG-D. The best-performing EDLC is N3DG, with high sp^2^ carbon. It also has fewer defects and reveals the highest nitrogen content, which could have created numerous active sites attracting the electrolyte. The latter causes faster infiltration and higher capacitance. Figure 6b presents the CV of N3DG at different scan rates from 100 mV/s to 1000 mV/s. Rectangular profile is maintained at all scan rates, which confirms the high rate capability of N3DG [33].

An Electrochemical Impedance Spectroscopy (EIS) comparison of the three types of graphene electrodes is given in Figure 6c. The imaginary and real impedances are plotted over a frequency range from 100 kHz to 0.1 Hz. The point where the EIS curve of each graphene type intersects the X-axis determines the Equivalent Series Resistance (ESR). This parameter represents the total resistance, including carbon-based electrode components, contact resistance at the interface, and the internal resistance of the electrolyte ions. The ESR is very low for all three graphene types, revealing values of 1.5 Ω, 2.0 Ω, and 2.2 Ω for N3DG-D, N3DG, and P3DG, respectively, which indicates lower dissipation of the stored energy, greater efficiency, and higher power performance. Following the EIS curves from the crosspoints determining the ESR and upwards, it is noticeable first a short 45° line, which is then extended to a 90° line parallel to the Y axis [21]. A short 45° line and its quick directional transition are due to the availability of active sites that can be reached quickly by the electrolyte, resulting in high-power capacitive behavior.

To better understand the electrochemical behavior, galvanostatic charge-discharge tests were conducted on the three electrodes (Figure 6d–f). All the electrodes revealed an isosceles triangle profile indicating EDLC behavior. The nitrogen-doped graphene types (N3DG and N3DG-D) showed slight non-linearity in their discharge curves, which could be attributed to the pseudo-capacitance due to oxygen and nitrogen functional groups confirmed by XPS [34]. During the GCD test, the current density was gradually increased, the discharge current and the discharge time were noted, and gravimetric capacitance was calculated [21,22]. The calculated values aligned with the CV profiles. N3DG exhibited the best gravimetric capacitance of 6.1 mF/g, followed by P3DG and N3DG, giving 1.74 mF/g and 0.32 mF/g, respectively, at a current density of 2 A/g (Table S1). Therefore, N3DG was confirmed to be superior due to its high sp^2^, low defect structure, and higher N-doping percentage than the other two forms of graphene.

The above results agree with our recent publication related to functionalizing N3DG through atmospheric pressure oxygen plasma treatment [20]. This procedure introduced oxygen functional groups like the carbonyl group onto the N3DG lattice, which already had hydroxyl, pyridinic, and pyrrolic nitrogen linkages. Such a functional plurality provided higher gravimetric capacitance, energy density, and cyclic stability [20]. The obtained results are also in agreement with the recent studies, which confirm that higher capacitance is primarily due to the oxygen functional groups like carbonyl bonds connected to pyridine-like structures. The latter can effectively improve the electrochemical activity of nanocarbon in addition to the hydroxyl group, which provides surface wettability, an attribute instrumental in electrochemical reactions [6,13].

## 4. Conclusions and Future Perspective

By comparing the electrochemical performances of the three types of graphene electrodes for EDLC application and lead detection, it was found that N3DG emerged superior to both P3DG and N3DG-D. We believe that the study confirms the positive electrochemical effects of high sp^2^, low defect structure, and the synergy between pyridinic nitrogen linkage and carbonyl/hydroxyl-based oxygen functional groups. From a future perspective, N3DG-based, flexible, microelectrode-type lead sensors capable of ppb resolution for lead detection can be fabricated and tested. As for the supercapacitor application, high-performing N3DG hybrid capacitors can be created by combining nitrogen-doped 3D graphene with battery materials to achieve high energy and power densities.

## Figures and Tables

**Table 1:** Synthesis details of P3DG, N3DG and N3DG-D.

Type of Graphene	Carbon Precursor	Dopant	Temperature
Pristine graphene (P3DG)	Methane	NA (Not Applicable)	1000 °C
Nitrogen-doped graphene (N3DG)	Methane	Acetonitrile	1000 °C
Nitrogen-doped graphene-Defective (N3DG-D)	Acetonitrile	Acetonitrile	750 °C

**Table 2:** Elemental Analysis.

Element (%)	Samples		
	P3DG	N3DG	N3DG-D
Carbon (C1s)	98.5	89.37	79.1
Oxygen (O1s)	1.5	8.63	20.2
Nitrogen (N1s)	-	2.0	0.7

**Table 3:** Active Areas and Peak Separation of Graphene Electrodes.

Electrodes	Peak Separation (mV)	Active Area (cm^2^)
P3DG	216	10.5
N3DG	318	175
N3DG-D	355	2.52

**Table 4:** Peak Height and Peak Area for N3DG and P3DG in a 0.1 M acetate buffer solution and 1.5 μM Pb^2+^ (number of trails for each electrode, *n* = 3).

Electrodes	Peak Height (μA)	Peak Area (V·μA)
N3DG	9.44 ± 1.0	0.77 ± 0.15
P3DG	3.83 ± 0.20	0.28 ± 0.06

## Data Availability

Data are contained within the article and Supplementary Materials.

## Supplementary Materials

The following supporting information can be downloaded at https://www.mdpi.com/article/10.3390/nano14010108/s1. Figure S1: Current density vs. Potential for N3DG-D, N3DG, and P3DG in 50 M KCl; Figure S2: Cyclic voltammetry of the electrodes tested in 2.5 mM Ruhex and 25 mM KCl (A) N3DG-D (B) N3DG and (C) P3DG; Figure S3: Electrochemical characterization of N3DG-D electrode (A) Voltammograms (B) Current vs. square root of scan rate (C) Current vs. scan rate (*n* = 3); Figure S4: Electrochemical characterization of N3DG electrode (A) Voltammograms (B) Current vs. square root of scan rate (C) Current vs. scan rate (*n* = 3); Figure S5: Electrochemical characterization of P3DG electrode (A) Voltammograms (B) Current vs. square root of scan rate (C) Current vs. scan rate (*n* = 3); Figure S6: Voltammograms of electrodes in 0.1 M acetate buffer without lead (black) and with 1.5 μM Pb^2+^ (red) (A) N3DG-D (B) N3DG and (C) P3DG; Table S1: Comparison of the gravimetric capacitance of P3DG, N3DG and N3DG-D electrodes.
