# Supplementary material for: Modified 3D Graphene for Sensing and Electrochemical Capacitor Applications
Source: Nanomaterials (Basel). 2024 Jan 2;14(1):108. doi: 10.3390/nano14010108 (PMC10780470; doi:10.3390/nano14010108)
Supplement: Supplementary file 1 [file nanomaterials-14-00108-s001.zip › nanomaterials-2748626-supplementary.pdf]

Supplementary Info

Modified 3D Graphene for Sensing and Electrochemical Capacitor Applications

Kavitha Mulackampilly Joseph [1], Gabrielle R. Dangel [2] and Vesselin Shanov [1,3,*]

[1] Department of Mechanical and Materials Engineering, University of Cincinnati, Cincinnati, OH 45221, USA; josephka@mail.uc.edu
[2] Department of Chemistry, University of Cincinnati, Cincinnati, OH 45221, USA; dangelgr@mail.uc.edu
[3] Department of Chemical and Environmental Engineering, University of Cincinnati, Cincinnati, OH 45221, USA
* Correspondence: vesselin.shanov@uc.edu

1. LEAD SENSING

1.1 Cyclic Voltammetry (CV)

Initial CV testing was done in 50 mM KCl from +0.1 V to -0.45 V, as shown in Figure S1. Due to the different geometrical areas of the electrodes, the currents were normalized using each electrode's active area. Then, the electrodes were tested in a Ruhex solution. Figures S2 (A-C) display the CV graphs for each electrode along with their respective blank CVs.

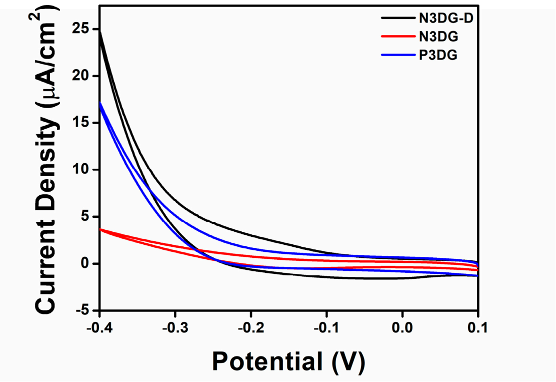

Figure S1. Current Density vs. Potential for N3DG-D (black), N3DG (red), and P3DG (blue) in 50 mM KCl.

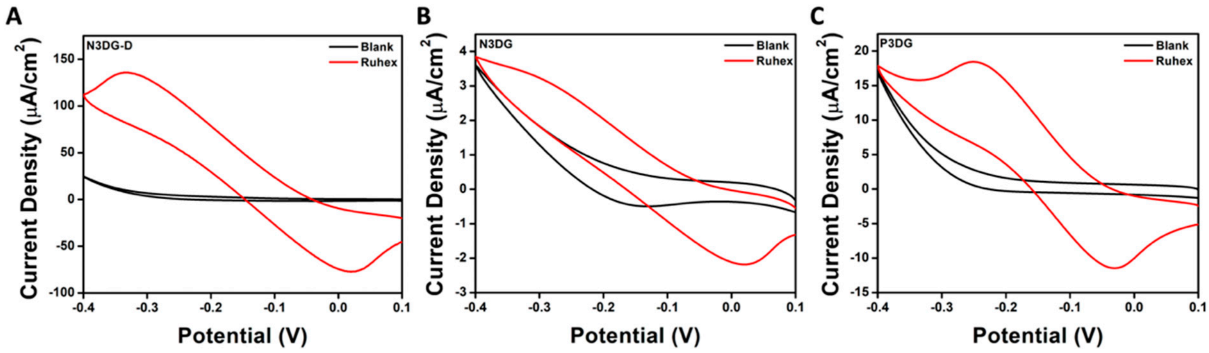

Figure S2. Cyclic voltammetry of the electrodes was tested in 2.5 mM Ruhex and 25 mM KCl using (A) N3DG-D, (B) N3DG, and (C) P3DG.

1.2 Scan Rate Study

In a solution of 2.5 mM Ruhex and 25 mM KCl, the electrodes were run at different scan rates varying from 0.001 to 0.2 V/s. Then, the cathodic and anodic peak currents were plotted versus the scan rate and square root of the scan rate. In the case of all electrodes, current versus the square root of the scan rate was linear, meaning all electrodes have diffusion-controlled electron transfer. Figure S3 shows the results for the N3DG-D electrode, Equations S1 and S2 suggest the linear relationship. Figure S4 and Equations S3 and S4 represent the data obtained for N3DG electrodes. Figure S5 and Equations S5 and S6 are for P3DG electrodes.

1.2.1 N3DG - D Electrode

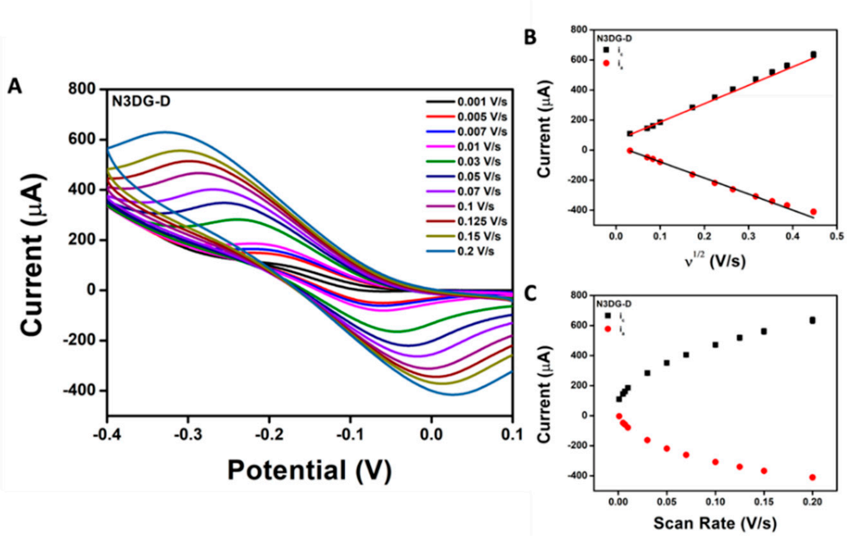

Figure S3. Electrochemical characterization of N3DG-D electrode. (A) voltammograms; (B) current vs. square root of scan rate; (C) current vs. scan rate (n=3)

$i_{pc} = [(122.5 \pm 3.3) \cdot 10^{-1}]\nu^{\frac{1}{2}} + (64.0 \pm 3.3)$ (Equation S1) $R^2 = 0.992$

$i_{pa} = [(-107.1 \pm 1.7) \cdot 10^{-1}]\nu^{\frac{1}{2}} + (30.0 \pm 2.1)$ (Equation S2) $R^2 = 0.997$

1.2.2 N3DG Electrode

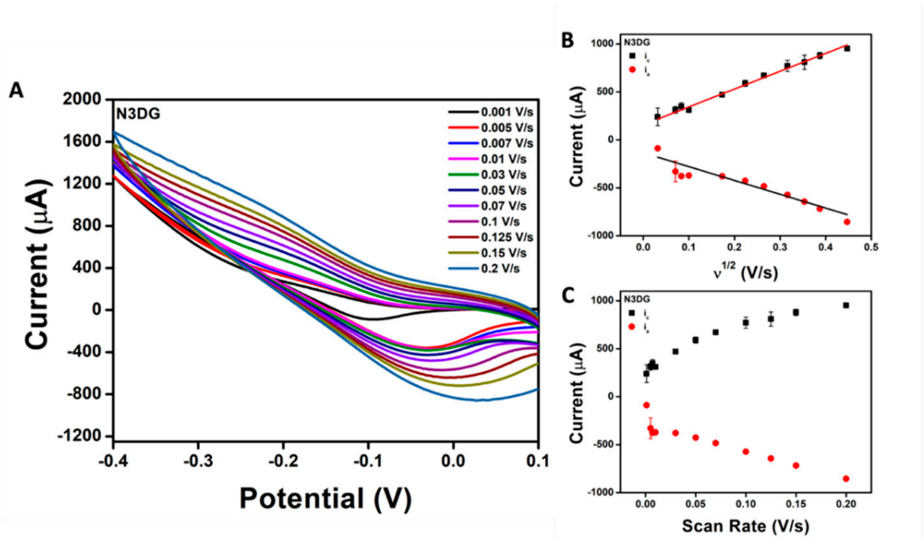

Figure S4. Electrochemical characterization of N3DG electrode. (A) voltammograms; (B) current vs. square root of scan rate; (C) current vs. scan rate (n=3)

$i_{pc} = [(185.9 \pm 7.5) \cdot 10^{-1}]\nu^{\frac{1}{2}} + [(15.7 \pm 1.9) \cdot 10^{-1}]$ (Equation S3) $R^2 = 0.984$

$i_{pa} = [(-144.1 \pm 15.3) \cdot 10^{-1}]\nu^{\frac{1}{2}} + [(-13.5 \pm 4.6) \cdot 10^{-1}]$ (Equation S4) $R^2 = 0.898$

1.2.3 P3DG Electrode

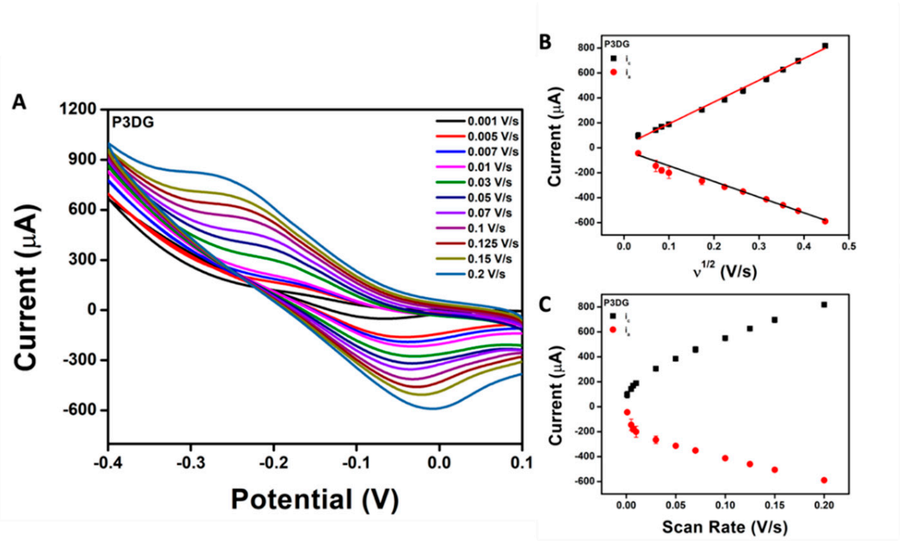

Figure S5. Electrochemical characterization of P3DG electrode. (A), voltammograms (B); current vs. square root of scan rate; (C) current vs. scan rate (n=3)

$i_{pc} = [(174.5 \pm 2.4) \cdot 10^{-1}]v^{\frac{1}{2}} + (18.0 \pm 2.2)$ (Equation S5) $R^2 = 0.998$

$i_{pc} = [(-125.6 \pm 3.6) \cdot 10^{-1}]v^{\frac{1}{2}} + [(-1.9 \pm 1.3) \cdot 10^{-1}]$ (Equation S6) $R^2 = 0.992$

1.3 Lead Detection

The electrodes were tested in 0.1 M acetate buffer (pH 4.5), where the black curves displayed in Figures S6 (A-C) were obtained before 1.5 μM Pb^{2+} was added. After adding Pb^{2+}, the curves were revealed in red. The data was baseline-corrected so the voltammograms could overlay each other. The full potential window of -1.2 V to +0.3 V is shown in these voltammograms. However, the lead peak in Figures S6 B and C is seen at around -0.5 V.

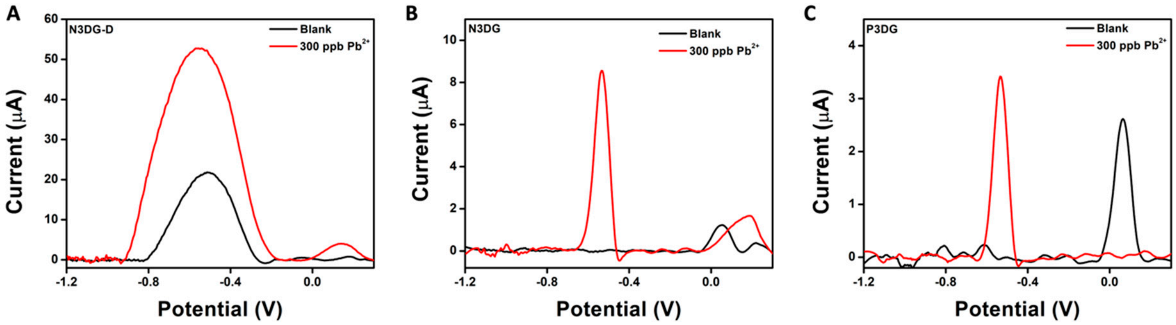

Figure S6. Voltammograms of the electrodes in 0.1 M acetate buffer without lead (black) and with 1.5 μM Pb^{2+} (red). (A) N3DG-D; (B) N3DG; and (C) P3DG.

2. EDLC CHARACTERIZATION

**Electrodes**	**Mass in mg**	**Discharge time (s)**	**Capacitance @ 2 A/g, at 1V**
P3DG	0.80	3.0	1.74 mF/g
N3DG	0.758	10.8	6.1 mF/g

N3DG-D	0.850	0.57	0.32 mF/g

Table S1. Comparison of gravimetric capacitance for P3DG, N3DG, and N3DG-D electrodes.
